# The genetic basis of tooth impaction: a systematic review

**DOI:** 10.1007/s00784-025-06520-0

**Published:** 2025-09-22

**Authors:** S. Papadopoulos, I. Ziakas, E. Panteris, A. Chatzigianni

**Affiliations:** 1 Faculty of Dentistry, School of Health Sciences, Aristotle University of Thessaloniki, Thessaloniki, Greece; 2 Biomic_AUTh, CIRI-AUTH Center for Interdisciplinary Research and Innovation, Aristotle University of Thessaloniki, Thessaloniki, Greece; 3 Department of Orthodontics, Faculty of Dentistry, School of Health Sciences, Aristotle University of Thessaloniki, Thessaloniki, Greece

**Keywords:** Tooth impaction, Impacted teeth, Genes, Genetics, Mutations

## Abstract

**Objectives:**

The aim of this review was to identify genes and genetic traits that cause tooth impaction by systematically collecting the best available evidence.

**Materials and methods:**

Relevant literature was searched on 10 databases up to March 2025 and search criteria were formulated using the PECOS and PRISMA guidelines. The eligibility criteria included case–control, cohort and cross-sectional observational studies, which examined human subjects regardless of age or sex and focused on the analysis of genes, alleles, gene variants, non-coding RNAs, or other genetic factors that cause tooth impaction. The risk of bias of eligible studies was evaluated using the Joanna Briggs Institute (JBI) Critical Appraisal Checklists.

**Results:**

Overall, 15 studies met the inclusion criteria. Several important genes were highlighted and mutations and polymorphism in these genes showed an increase in the risk of tooth impaction. Specifically, MSX1 rs12532, PAX9 rs4904210 and rs2073247, AXIN2 rs2240308, as well as MSX2 rs4868444 and ARNT2 rs140220410 revealed significant association with canine, third molar or general tooth impaction. Synergistic effects of some gene genotypes were also addressed. The third molar impaction was associated with specific long non-coding RNAs of the corresponding dental follicles, and with the individual’s blood group. According to the JBI checklist, the studies showed high methodological quality.

**Conclusion:**

Three key genes, namely MSX1, PAX9 and AXIN2, appear to have an important role in tooth impaction. Moreover, limited evidence suggests that long non-coding RNAs and the type of individual’s blood group could be potential biomarkers of tooth impaction. However, the contradictory results from the included studies reduce the certainty of any solid conclusions. Additional studies with large samples involving advanced methodologies for genetic testing are essential to pinpoint the underlying genetic factors of tooth impaction.

**Clinical relevance:**

Understanding the genetic factors and specific mutations behind tooth impaction, may enhance early diagnosis, prevention of impaction and treatment.

**Supplementary Information:**

The online version contains supplementary material available at 10.1007/s00784-025-06520-0.

## Introduction

Tοοth impaction is a common dental anomaly characterized by the failure of teeth to erupt into their proper functional positions in the dental arch. According to Baccetti et al. [1] the maxillary permanent canine has the highest frequency of impaction following the third molar, becoming impacted in 0,8–5,2% of the general population with more than twice the chance to happen compared to the mandibular canine [1]. Female patients are affected 2 to 3 times more than males and the ratio of palatal to buccal impaction is 8 to 1. Based on Alalola et al. [2] following maxillary canine impaction, the mandibular canine had an impaction rate of 0,18% followed by the mandibular premolars of 0,08%. Impacted teeth can cause various clinical problems, including pain, infection, and damage to adjacent teeth roots and bone. Understanding the genetic factors underlying teeth impaction is crucial for developing preventive and therapeutic strategies [3–6].

The exact etiology of tοοth impaction remains unknown. Two theories have been proposed according to the literature, the “guidance theory” and the “genetic theory” [7, 8]. According to the “guidance theory” tooth impaction occurs when there is a disruption in the natural path that guides a tooth to its proper position in the mouth. Becker et al. [8] has mentioned that local obstructions such as primary canine retention, supernumerary teeth, odontoma, abnormalities of the central-lateral incisor and first premolar or local pathology and dental agenesis lead to maxillary canine impaction.

Based on the “genetic theory” mutations in key developmental genes cause tooth impaction alongside with other genetic features like dental agenesis, spaced dentitions and peg-shaped teeth. Ultimately the impaction is just another corresponding inherited characteristic as for this theory [7–14]. Especially for the palatal impacted canine, both theories agree on the fact that specific genetic features appear alongside its palatal displacement. Even though the agenesis of the lateral incisor has genetic proneness [15, 16], Papageorgiou et al. [17] states the probability of palatally impacted canines as a multifactorial condition. Through this study the authors clarify that the maxillary lateral incisor absence causes mostly palatal canine displacement and often impaction due to guidance problems. Other recent studies have highlighted the involvement of several genes in dental development and impaction [18, 19]. Notably, the PAX9, MSX1, AXIN2, and IRF6 genes have been implicated in tooth development and tooth anomalies [20–22]. These genes play critical roles in the regulation of tooth morphogenesis and eruption [23, 24]. Variations in these genes, including polymorphisms and haplotypes, may contribute to the susceptibility to teeth impaction [25–27]. Additionally, non-coding RNAs, particularly long noncoding RNAs (lncRNAs) have been shown to be differentially expressed in dental follicle tissues of impacted third molars and promote osteogenic differentiation [28, 29]. These RNAs may have regulatory functions in gene expression and cellular processes involved in tooth impaction [30, 31].

This systematic review aims to synthesize the current evidence on the genetic factors associated with tooth impaction. By consolidating the findings from various studies, this review seeks to provide a comprehensive understanding of the genetic determinants of teeth impaction and identify potential targets for future research and clinical interventions.

## Materials and methods

### Protocol and registration

The protocol for this systematic review was developed in advance following the guidelines outlined in the Cochrane Handbook for Systematic Reviews of Interventions [32]. This review adheres to the PRISMA guidelines [33] and its extension for abstracts [34] (PROSPERO Registration Number: CRD42024597963. Initial and revised protocol can be found at https://www.crd.york.ac.uk/PROSPERO/view/CRD42024597963).

### Information sources and search strategy

Ten electronic databases i.e. MEDLINE, Scopus, Web of Science, Google Scholar, Cochrane Library, Science Direct, Ovid, ProQuest, Virtual Health Library, Clinical Trials Gov. were systematically and unrestrictedly searched up to April 2024 and updated in March 2025. Appropriate MeSH terms and related keywords were tailored for each database (Appendix, Table A1). The search strategy did not impose any restrictions on language, publication date, or publication status. Translation tools were used in studies that were conducted in languages other than English.

### Eligibility criteria and study selection

The eligibility criteria were established in advance. The PECOS (Population, Exposure, Comparison, Outcome, and Study Design) framework was used (Table 1). A study was deemed eligible if it included at least one gene related to impacted teeth and met all inclusion criteria while avoiding any exclusion criteria.

**Table 1:** Inclusion and exclusion criteria as defined by PECO framework

PECO	Inclusion criteria	Exclusion criteria
Participants	• Humans of any age or gender • Individuals with impacted teeth, provided they do not have significant craniofacial syndromes or other systemic disorders known to affect tooth eruption • Otherwise, healthy participants when collecting necessary comparative data	• Animal or in vitro studies • Patients with craniofacial syndromes or other systemic conditions known to influence tooth development and eruption • Studies involving only patients with unrelated medical conditions
Exposure (E)	Presence of impacted tooth/teeth (including mandibular third molars, maxillary canines, or other impacted permanent teeth)	No data on impacted teeth or no clear definition of tooth impaction in the study
Comparison (C)	Participants without impacted teeth (if the study design includes a control group), or a baseline/control group used for genetic comparison	No control or comparator group in designs that specifically require one
Outcome (O)	Genetic factors related to impacted teeth, such as: • SNPs (single nucleotide polymorphisms) • Specific alleles or gene variants • Gene expression levels • Blood-group associations or other inherited characteristics	• Studies without any genetic data related to tooth impaction • Outcomes not relevant to genetic risk or blood-group associations
Study Design	• Observational studies (cohort, case–control, cross-sectional) that assess genetic factors in patients with and without impacted teeth • Prospective or retrospective designs are acceptable if they report primary data on genetics	• Randomized clinical trials • Non-randomized clinical trials • Articles studying supernumerary teeth, tooth agenesis, mesiodens, odontomas, etc • Editor's choices • Unsupported expert opinions • Replies to the author/editor • Interviews • Commentaries • Books/conference abstracts • Summaries • Case series without a control group • Case reports or reports of cases • Narrative reviews • Systematic reviews • Meta-analyses

All papers were collected from each database. Subsequently, duplicates were removed and the remaining articles were screened based on title and abstract by the first two authors (SP, IZ). Afterwards, the full texts were evaluated by both authors to decide upon each paper’s eligibility. The study selection procedure was conducted independently and double-blind for both authors. In cases where no consensus between the two authors was available, consultation was sought by a senior author (AC). If a trial was published in multiple languages, the English version was given preference. Agreement on the inclusion of articles by both raters was determined using Cohen's Kappa (κ).

### Data collection process and data items

Details on study characteristics were recorded, and data extraction process of each study was registered on predefined extraction forms. In case of absent information, contact with the corresponding authors was attempted via electronic communication. Reports related to the same trial or patient cohort were combined. Data collection was carried out independently by two authors (SP, IZ). Any discrepancies were resolved through consultation with the senior author.

Studies providing data solely on environmental factors were excluded from the evaluation of genetic influence, as these methods do not reliably differentiate between genetic and environmental contributions to tooth impaction. If specific genetic traits were clearly reported in the included articles, they were categorized accordingly. In the absence of direct data, the classification was based on the presence of known genetic markers associated with impacted teeth. Gene-related factors to be analyzed may include 1) specific genes or genetic variants studied (e.g., PAX9, MSX1), and 2) the type of genetic analysis performed (e.g., genome-wide association study versus targeted gene analysis) 3) Blood-groups or other hereditary characteristics.

### Risk of bias assessment

The Joanna Briggs Institute's (JBI) critical appraisal checklist was selected for assessing the risk of bias of case–control and cross-sectional studies.

In the case of sufficient number of eligible studies and adequate data, meta-analysis would be performed.

## Results

### Study selection

**Fig. 1:**
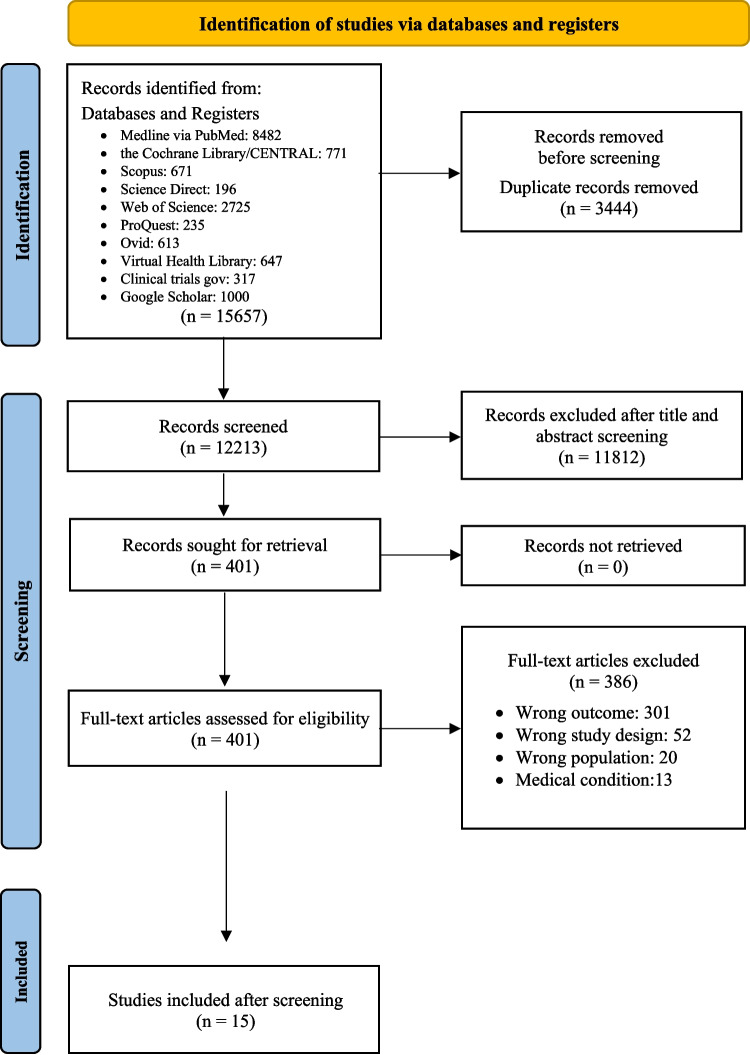
Flow chart showing the selection of studies performed according to the PRISMA guidelines

As presented in Fig. 1, 15657 records were initially identified via systematic and hand search. After removal of duplicates, 12213 remained and were screened by title, abstract and full texts based on the eligibility criteria. Lastly, 15 articles remained [20, 28, 35–47] and were included in the systematic review for further analysis to evaluate the genetic associations of various genes, single nucleotide polymorphisms (SNPs) and other hereditary characteristics with dental anomalies, particularly focusing on tooth impaction. During this procedure the two authors disagreed on 30 papers, giving an observed agreement, Po, of 91.5% with Cohen’s Kappa = 0.83, a high value indicating good concordance between the authors.

### Study characteristics

The sample sizes of the included studies varied widely, ranging from 11 participants in a study by Uribe et al. [39] to 3,579 participants in a study by Alotaibi et al. [35]. The age of participants also varied across studies, with the sample presenting a mean age of 45,5 years. After evaluation of the included studies, these were found to primarily involve patients with dental anomalies, such as tooth impaction. Cross-sectional designs were the most common study type, as seen in nine of the included studies [28, 39–43, 45–47]. The studies employed a variety of methodologies, including genome-wide association studies (GWAS), Real-Time Polymerase Chain Reaction (RT-PCR) and others.

The sample characteristics, type of study, methodology and outcomes of each included study regarding genes and variants related to impaction are presented in detail in Tables A2 and A3 (Appendix). The excluded studies are listed in Table A4.

### Risk of bias assessment and additional analyses

According to the JBI checklist, most of the studies showed high methodological quality. The risk of bias of the included studies is presented in Tables 2 and 3.

**Table 2:** Risk of bias of the included studies by the JBI critical appraisal checklist for cross-sectional studies

Checklist Items	Were the criteria for inclusion in the sample clearly defined?	Were the study subjects and the setting described in detail?	Was the exposure measured in a valid and reliable way?	Were objective, standard criteria used for measurement of the condition?	Were confounding factors identified?	Were strategies to deal with confounding factors stated?	Were the outcomes measured in a valid and reliable way?	Was appropriate statistical analysis used?
Barbato et al. [43]	Yes	Yes	Yes	Yes	Unclear	No	Yes	Yes
Trakinienė et al. [40]	Yes	Yes	Yes	Yes	Yes	Yes	Yes	Yes
Uribe et al. [39]	Yes	Yes	Yes	Yes	yes	No	Yes	Yes
Olsson et al. [42]	Yes	Yes	Yes	Yes	Unclear	Unclear	Yes	Yes
Ahmadi et al. [46]	Yes	Yes	Yes	Yes	Unclear	No	Yes	Yes
Bozgeyik et al. [41]	Yes	Yes	Yes	Yes	Yes	No	Yes	Yes
Ege et al. [28]	Yes	Yes	Yes	Yes	Unclear	Unclear	Yes	Yes
Alassaf et al. [47]	Yes	Yes	Yes	Yes	Unclear	No	Yes	Yes
Almalki et al. [45]	Yes	Yes	Yes	Yes	Unclear	No	Yes	Yes

**Table 3:** Risk of bias of the included studies by the JBI critical appraisal checklist for case–control studies

Checklist items	Were the groups comparable other than the presence of disease in cases or the absence of disease in controls?	Were cases and controls matched appropriately?	Were the same criteria used for identification of cases and controls?	Was exposure measured in a standard, valid and reliable way?	Was exposure measured in the same way for cases and controls?	Were confounding factors identified?	Were strategies to deal with confounding factors stated?	Were outcomes assessed in a standard, valid and reliable way for cases and controls?	Was the exposure period of interest long enough to be meaningful?	Was appropriate statistical analysis used?
Devi and Padmanabhan [38]	Yes	Yes	Yes	Yes	Yes	Yes	Yes	Yes	Yes	Yes
Vitria et al. [37]	Yes	Yes	Yes	Yes	Yes	Unclear	Yes	Yes	Yes	Yes
Adeyemo et al. [44]	Yes	Yes	Yes	Yes	Yes	Yes	Yes	Yes	Yes	Yes
Alotaibi et al. [35]	Yes	Yes	Yes	Yes	Yes	Yes	Yes	Yes	Yes	Yes
Trybek et al. [36]	Yes	Yes	Yes	Yes	Yes	Yes	Yes	Yes	Yes	Yes
Trybek et al. [20]	Yes	Yes	Yes	Yes	Yes	Yes	Yes	Yes	Yes	Yes

A meta-analysis was not performed as initially planned, due to the heterogeneity in genetic markers, outcome definitions and study populations. In cases where studies assessed similar variants, data were still insufficient or inconsistent to allow valid pooling.

### Genetic associations and results

Of the included studies, eight focused specifically on third molar impaction [28, 40–42, 44–47], while three investigated maxillary canine impaction [37, 38, 43]. Additionally, three studies explored tooth impaction more generally [20, 36, 39], and one study examined craniofacial morphogenesis in relation to tooth development [35].

A total of 16 genes and numerous SNPs were identified across the studies. The most frequently studied genes included MSX1, PAX9, and AXIN2, all of which are implicated in craniofacial development and dental anomalies.

MSX1 was examined in four studies [20, 36, 38, 42], with rs12532 and rs8670 being the most common SNPs analyzed.

### Genetic variants and tooth impaction

Trybek et al. [36] focused on the MSX1 rs12532 polymorphism and observed a notable link between the A allele and severe tooth impaction, particularly in cases involving more than three impacted teeth compared to those with fewer impacted teeth (*p* = 0.009), while MSX1 rs8670 did not show significant differences between groups. This underscores the impact of genetic variations in MSX1, a key gene in odontogenesis, on dental eruption patterns.

PAX9 has been also frequently studied, particularly rs4904210. Specifically, Trybek et al. [20] reported associations of this allele with tooth impaction. In the study of Vitria et al. [37], the TT genotype of PAX9 rs4904210 and rs12881240 were associated with an increased risk of maxillary canine impaction. However, while the study identified potential genotypic risks, statistical significance was not achieved. Despite this limitation, the findings add to the understanding of PAX9’s role in regulating precise tooth positioning.

AXIN2 was highlighted for its role in tooth impaction, with significant results found for rs2240308 according to the study by Trybek et al. [20].

The study by Alotaibi et al. [35] examined genetic variants, such as MSX2 rs4868444 and ARNT2 rs140220410, which play a role in craniofacial morphogenesis. They identified a significantly higher prevalence of tooth impaction among unaffected relatives of orofacial cleft (OFC) patients, with 80.49% compared to 19.51% in general population (*p* = 0.01). This highlights the genetic predisposition to dental anomalies and emphasizes the influence of subtle familial traits on dental health.

Devi and Padmanabhan [38] investigated the synergistic effects of MSX1 rs12532 and PAX9 rs2073247. Their study revealed that individuals carrying both polymorphisms had a higher risk of palatal impaction of maxillary canines, demonstrating a synergistic interaction between these genes. This interplay suggests that MSX1 and PAX9 may collaboratively regulate key developmental pathways, such as Wingless-Type (WNT) and Fibroblast Growth Factor (FGF) signaling, which are crucial for proper tooth eruption.

In more current studies, Adeyemo et al. [44] examined the contribution of several SNPs in the impaction of mandibular third molars. The rs6504591 G/T variant in the WNT9B gene appeared to confer a twofold increased risk of impaction, however, the association did not reach statistical significance.

Two interesting studies [28, 41] focused on the expression profiles of selected long non-coding RNAs (lncRNAs) in dental follicle (DF) tissues associated with asymptomatic impacted mandibular third molars. The findings indicate that specific lncRNAs, particularly MEG3 and NORAD [28], may be actively involved in fundamental biological processes, including osteogenic differentiation, DNA damage response, and the early phases of odontogenic transformation. These molecular insights underscore the potential diagnostic value of lncRNAs as biomarkers in the clinical evaluation of DF tissues, particularly in cases where radiographic evidence of pathology is absent.

### ABO blood groups and tooth impaction

Three recent studies have offered interesting insights into how ABO blood groups might subtly influence the occurrence of impacted third molars, hinting at an indirect genetic link. Ahmadi et al. [46] found no correlation between ABO groups and third molar impactions. In agreement with the above, the retrospective analysis by Alassaf et al. [47] found no association between blood type and third molar impaction. On the contrary, the study of Almalki et al. [45] observed notable differences in tooth impaction rates among Saudi adults with different blood groups, raising the possibility that genetic factors determining ABO types could also contribute to dental anomalies, adding weight to the suggestion that inherited blood group traits may subtly affect the likelihood or characteristics of impacted teeth. Although none of these studies explicitly detail genetic mechanisms, the most recent one adds weight to how inherited traits like blood groups could indirectly shape dental health outcomes.

It is worth mentioning that within the included studies, some results were not positive on the influence of genetics on tooth impaction [42, 43] other implied an indirect connection [35, 37, 39, 42], while others found marginally significant associations [44].

## Discussion

The process of tooth development is especially complicated and is strongly associated with a network of genetic signals. Dental anomalies during tooth eruption are believed to be caused by numerous reasons, such as genetic, epigenetic and environmental [48, 49]. In this systematic review, we have pinpointed three key genes - MSX1, PAX9 and AXIN2 - which play a symbolic role in tooth development and specifically in tooth impaction. These genes not only influence tooth formation and eruption, but also play a broader role in craniofacial development and dental patterning anomalies [50].

Importantly, mutations in PAX9 and MSX1 have been strongly linked to tooth agenesis, particularly hypodontia and oligodontia; PAX9 is linked to posterior tooth agenesis, while MSX1 is more frequently associated with anterior tooth agenesis [51–55]. These genotype–phenotype patterns suggest that certain genes may exert regional influences within the dental arch, which could explain the observed patterns of impaction depending on the location and number of missing teeth [56–58]. For instance, absence of a permanent tooth may disrupt the eruption pathway of neighboring teeth, indirectly contributing to impaction. Mostowska et al. [23] mention that individuals with mutations in MSX1 have higher probability of tooth agenesis and impacted third molars.

Furthermore, these genetic alterations may result in craniofacial and dental morphological anomalies, such as peg-shaped or diminutive lateral incisors [59], which could present guidance issues during eruption or pose anatomical obstructions, ultimately leading to impaction [17, 60, 61]. This highlights the importance of evaluating not only tooth number, but also tooth morphology and jaw architecture in patients with suspected genetic dental anomalies.

The findings also offer support for broader theoretical frameworks, such as Peck's Dental Anomaly Pattern (DAP), which combines delayed eruption, impaction, and agenesis under a common etiologic category [62]. From this perspective, genetic mutations in MSX1, PAX9, and AXIN2 may represent a common etiological thread, potentially unifying these clinical presentations. In a study examining patterns of dental agenesis, the presence of causative mutated genes was highlighted, showing that isolated dental agenesis can exist as part of a spectrum of other conditions [63].

Recent findings are helping to expand how we think about third molar impaction, by introducing a wider range of possible genetic and epigenetic influences. Traditional research has mostly concentrated on clear genetic mutations in specific genes, such as MSX1, PAX9, and AXIN2, which are directly linked to tooth development. However, the work by Adeyemo et al. [44] suggests that genes related to general body growth - like those that affect height - might also play a part in when and how teeth erupt. This opens the possibility that traits not strictly tied to the oral environment and jaw development may still influence dental outcomes in indirect ways.

On the same note, non-coding RNAs seem to have a role as well with the expression profiles of selected long non-coding RNAs in dental follicle tissues associated with asymptomatic impacted mandibular third molars [28].

The group of studies looking at ABO blood types [45–47] add another interesting perspective. Even though these studies have contradictory results, one of them found that people with certain blood groups were more likely to have impacted teeth. Since blood type is inherited, this points to a possible, even if indirect, genetic connection, even though not any specific genes involved in impaction were identified in those investigations.

Altogether, these studies show that tooth impaction is not solely determined by a single cause. There is high probability that the result of many overlapping factors - some genetic, some related to how genes are controlled, and others linked to overall physical traits. To better understand the causes and improve prevention or treatment of impacted teeth, future research will need to look at the full range of genetic and biological influences, rather than focus on one area alone.

## Limitations

This systematic review has several limitations. Firstly, the studies included demonstrated considerable heterogeneity regarding sample sizes, methodologies, genetic analyses performed, and populations studied, potentially influencing the consistency and comparability of findings. Additionally, the relatively small number of studies available for certain genes and polymorphisms restricts the ability to generalize the results and draw definitive conclusions regarding their role in tooth impaction. Future research with more uniform methodologies, larger sample sizes, and comprehensive genetic analysis is essential to overcome these limitations and confirm the genetic basis of tooth impaction.

## Conclusion

The results of this systematic review emphasize the role of genetic factors in the etiology of tooth impaction. Key genes, such as MSX1, PAX9, and AXIN2 appear to play a significant role in the development of tooth impaction. Limited evidence suggests that long non-coding RNAs and the type of individual’s blood group could also be potential biomarkers of tooth impaction. However, the results are contradictory, with some studies showing strong associations, whilst others showing no statistically significant correlations of tooth impaction with genetic traits. The heterogeneity of study designs, sample sizes, and patient populations limits the ability to draw firm conclusions. Further research with larger cohorts and advanced methodologies is warranted to clarify the genetic basis of tooth impaction.

## Supplementary Information

Below is the link to the electronic supplementary material.

Supplementary file1 (DOCX 117 KB)

Supplementary file2 (DOC 32 KB)

## Data Availability

Data of this study is available upon reasonable request.
