# Supplementary material for: The genetic basis of tooth impaction: a systematic review
Source: Clin Oral Investig. 2025 Sep 22;29(10):469. doi: 10.1007/s00784-025-06520-0 (PMC12454535; doi:10.1007/s00784-025-06520-0)
Supplement: Supplementary file 1 — Supplementary file1 (DOCX 117 KB) [file 784_2025_6520_MOESM1_ESM.docx]

**APPENDIX**

**Table A1:** Search strategy for each database.

| **Electronic databases** | **Search strategy** | **Limits** | **Hits** | **Search date (updated)** |
| --- | --- | --- | --- | --- |
| **MEDLINE**  Searched via PubMed | (genes OR gene OR genet* OR geno*) AND (tooth OR teeth OR dent*) AND (impaction OR impacted) | no | 8482 | March 2025 |
| **Scopus** | (genes OR gene OR genet* OR geno*) AND (tooth OR teeth OR dent*) AND (impaction OR impacted) | title, abstract, keywords | 671 | March 2025 |
| **Web Of Science** | (genes OR gene OR genet* OR geno*) AND (tooth OR teeth OR dent*) AND (impaction OR impacted) | text, title, abstract | 2725 | March 2025 |
| **Google Scholar** | (impacted OR impaction) AND (teeth OR tooth) | title | 1000 | March 2025 |
| **Cochrane CENTRAL register of controlled trials** | (genes OR gene OR genet* OR geno*) AND (tooth OR teeth OR dent*) AND (impaction OR impacted) | no | 771 | March 2025 |
| **Science Direct** | (genes OR gene OR genet OR geno) AND (tooth OR teeth OR dent) AND (impaction OR impacted) | title, abstract, keywords | 196 | March 2025 |
| **Ovid** | (genes OR gene OR genet* OR geno*) AND (tooth OR teeth OR dent*) AND (impaction OR impacted) | abstract | 613 | March 2025 |
| **ProQuest** | (genes OR gene OR genet* OR geno*) AND (tooth OR teeth OR dent*) AND (impaction OR impacted) | text, abstract | 235 | March 2025 |
| **Virtual health**  **library** | (genes OR gene OR genet* OR geno*) AND (tooth OR teeth OR dent*) AND (impaction OR impacted) | no | 647 | March 2025 |
| **Clinical Trials Gov** | impacted AND (teeth OR tooth) | no | 317 | March 2025 |
| ***Sum*** | | | 15657 | March 2025 |

**Table A2:** Study characteristics of the included studies.

| **Ref/Year/Authors** | **Type of Study** | **Number of patients** | **Age (Years)**  **Mean (Range)** | **Diagnostic Criteria of tooth impaction** |
| --- | --- | --- | --- | --- |
| Barbato et al. (2018) [43] | Cross-sectional | 14 (3 males, 11 females) | 13-83 yrs  Mean age = 48 | Diagnosis was based on panoramic radiographs, oral photographs, anamnestic data and oral examination. |
| Trakinienė et al. (2018) [40] | Cross-sectional | 212 twins (80 dizygotic, 132 monozygotic) | Dizygotic-twin group,  Mean age = 18 ± 0.56  Monozygotic-twin group,  Mean age = 19 ± 0.74 | Diagnosis was based on panoramic radiographs and lateral cephalograms. |
| Uribe et al. (2018) [39] | Cross-sectional | 11 (6 females, 5 males) | 10-16 yrs  Mean age = 13 | Impaction was decided when eruption was considered delayed, and for which there was clinical and radiographic evidence that further eruption may not occur within the normal period of growth. |
| Devi & Padmanabhan (2019) [38] | Case-control | 100 (study group: 50, control group: 50, 66 females, 34 males) | 11-35 yrs  Mean age = 23 | Diagnosis was based on the technique suggested by Stanley Jacobs (Jacobs, 1999). CBCT scans were taken when required to confirm diagnosis. |
| Vitria et al. (2019) [37] | Case-control | 121 (study group:83, control group: 38, 68 females, 53 males) | 10-25 yrs  Mean age = 17.5 | Diagnosis was based on clinical examinations and radiographic interpretations performed by radiologists and orthodontists. |
| Olsson et al. (2020) [42] | Cross-sectional | 32 (18 females, 14 males) | N/A | Diagnosis was based on orthopantomography with which the Pell and Gregory’s classification and the Winter’s classification were used. |
| Adeyemo et al. (2021) [44] | Case-control | 400 (study group:200, control group: 200, 217 females, 183 males) | Mean age = 24.2 | No methods were mentioned for diagnosis of tooth impaction. |
| Ahmadi et al. (2021) [46] | Cross-sectional | 320 (184 females, 136 males) | Mean age = 29 ± 6 | Clinical oral examinations, X-ray panoramic, and CBCT images investigation were undertaken to diagnose bony-impacted teeth. |
| Alotaibi et al. (2021) [35] | Case-control | 3,579 (1,392 no anomalies, 2,187 unaffected relatives of patients with OFC) | 8-82 yrs  Mean age = 31 | Diagnosis was based on oral examination and intraoral photos. Impaction was diagnosed only via canines that had not erupted even though permanent second molars had erupted. |
| Trybek et al. (2021) [36] | Case-control | 392 (study group: 204, control group: 188) | N/A | Diagnosis was based on panoramic radiographs and oral examination. |
| Bozgeyik et al. (2022) [41] | Cross-sectional | 42 (26 females, 16 males) | Mean age = 24.88 ± 5.133 | Diagnosis was based on panoramic radiographs. Patients with similar Winters, Pell- and - Gregory classification of impacted mandibular third molars were enrolled. |
| Ege et al. (2022) [28] | Cross-sectional | 30 (16 females, 14 males) | Mean age = 24.33 ± 5.02 | Diagnosis was based on panoramic radiographs. Patients with similar Winters, Pell- and - Gregory classification of impacted mandibular third molars were enrolled. |
| Alassaf et al. (2023) [47] | Cross-sectional | 856 (117 females, 739 males) | Mean age males = 34.78 ±13.68 | Diagnosis was based on panoramic radiographs, impacted third molars were classified based on angulation Mesioangular, Distoangular , Horizontal, or, Vertical. |
| Trybek et al. (2023) [20] | Case-control | 392 (study group: 204, control group: 188) | N/A | Diagnosis was based on panoramic radiographs and oral examination. An impacted tooth was defined as a tooth surrounded by bone in X-ray images but absent in the dental arch during eruption. |
| Almalki et al. (2024) [45] | Cross-sectional | 300 (113 females, 187 males) | Mean age females = 25.07 ± 2.03  Mean age males = 25.02 ± 1.94 | Diagnosis was based on radiographic and oral examination using orthopantomography if a third molar was unerupted. |

**Table A3:** Extended Methods, genes, their variants and conclusions.

| **Ref/Year/Authors** | **Methods** | **Genes/**  **Blood groups** | **Variant rs** | **Results** | **Conclusions** |
| --- | --- | --- | --- | --- | --- |
| Barbato et al. (2018) [43] | 1) Whole exome sequencing was done in individual members of a single family.  2) Selected candidate variants were further analyzed by Sanger Sequencing.  3) Variants were PCR-amplified by using GoTaq G2 Flexi DNA polymerase and custom primers.  4) Sanger sequencing was performed by using the ABI BigDye Terminator Sequencing Kit V.3.1 as per the manufacturer's protocol and an ABI Prism 3500 Genetic Analyzers.  5) Sequence electropherograms were analyzed by using ChromasPro V.1.7.5.  6) Segregation analysis was performed on all family members for whom DNA was available. | EDARADD  COL5A  RSPO4  T  NELL1 | rs114632254  rs61735045  rs6140807  rs117097130  rs141323787 | 1) 14 family members from which, 8 subjects were affected by canine anomalies: 4 by canine palatal impaction, either monolateral or bilateral, 2 by canine agenesis, either monolateral or bilateral, 2 by canine ectopic eruption, both monolateral.  2) Heterozygous variant in EDARADD rs114632254 was associated with tooth agenesis.  3) Affected Subjects III:1 and III:4, who showed a phenotype of impacted/ectopic erupted canines, shared missense heterozygous variants in RSPO4 rs6140807, T rs117097130, and NELL rs141323787 genes.  4) The COL5A1 variant rs61735045 which is associated with dental anomalies was found in the affected subjects III:5 and III:6, and unaffected subject III:2.  5) The RSPO4 variant rs6140807 was also present in subject III:3, who did not manifest any dental anomaly.  6) The T variant rs117097130 was also identified in unaffected subjects (III:2 and III:3).  7) The NELL rs141323787 was also expressed in subjects who did not exhibit any dental anomaly (III:2 and III:3). | Overall, the sample size is only 14 family members, which is a limitation. Other than the EDARADD rs114632254, COL5A1 rs61735045, RSPO4 rs6140807, T rs117097130, and NELL rs141323787 genes were present in both affected and unaffected patients, signalizing that there is no guaranteed involvement of them in dental anomalies like tooth impaction. |
| Trakinienė et al. (2018) [40] | 1) Determination of zygosity was performed with DNA tests with polymerase chain reaction (PCR) for the amplification of short tandem repeats and 15 specific DNA markers.  2) Skeletal maturity stage (CVM stage) was estimated using the lateral cephalograms.  3) Statistical analysis was performed with SPSS software. | No genes were mentioned. | No rs variant was mentioned. | 1) The eruption level of the maxillary third molars showed almost the same tendency between siblings in both dizygotic and monozygotic twins. However, the correlations were higher in the monozygotic twins. | Generally, the study mentions the importance of additive genetic factors in the size and eruption of teeth. |
| Uribe et al. (2018) [39] | 1) Immediately after their removal, the DF was placed in PAX gene tissue containers.  2) Genotyping took place with Real-Time PCR.  3) RNA extraction was carried out using the commercially available PAX gene Tissue RNA Kit.  4) Gene expression data were analyzed using the PRISM 7 software package. | RUNX2  OSX  ALP  OCN  CX43  BMP2  MCP1 | No rs variant was mentioned. | 1) Highest levels of expression were for genes involved in bone formation, (RUNX2 and CX43).  2) Relatively high gene expression levels were seen in all the samples for OSX, ALP, and OCN whereas the BMP2 gene showed very low expression in all the samples.  3)No significant difference was observed when comparing the follicles and relating their gene expression levels to their different clinical situations. | The genes RUNX2, OSX, ALP, OCN, CX43 contribute to the alveolar bone formation, which might have an influential role in the late pre-emerged tooth stage. |
| Devi & Padmanabhan (2019) [38] | 1) Genotyping was made by using polymerase chain reaction (PCR) and restriction fragment length polymorphism (RFLP).  2) The significance of the differences among the groups was assessed by odds ratio and Chi-squared test. | MSX1  PAX9 | rs12532  rs2073247 | 1) The MSX1 rs12532 and the PAX9 rs2073247 showed a statistically significantly higher association with palatal impaction of maxillary canines. Furthermore, the presence of AG/CT genotypes of these genes in a patient caused a significant increase in the risk for palatal impaction. | High importance of the MSX1 rs12532 and the PAX9 rs2073247 in palatal canine impaction. |
| Vitria et al. (2019) [37] | 1) Samples of genomic DNA were obtained from a buccal mucosa swab.  2) Genotyping was performed with polymerase chain reaction (PCR).  3) DNA from the PCR products was purified using a QIAquick PCR purification kit and purified DNA was sequenced by First-Base Laboratories.  4) Sequencing data were edited using BioEdit software and verified using NCBI BLAST.  5) Statistical analysis of the data was performed using SPSS. | PAX9  The fourth SNP in exon 3 maps to chromosome 14, position 36,666,530, and has not been previously reported. | rs375436662  rs12881240  rs4904210 | 1) TT genotype in PAX9 (rs12881240) showed a higher degree of association with maxillary canine impaction than those with the CC genotype or the CT genotype [odds ratio (OR): 2.61; 95% Confidence interval (CI): 0.29–23.61] and (OR: 1.28; 95% CI: 0.57–2.89), although these results were not statistically significant (*p > 0.05*).  2) No statistical difference in allele frequency was observed between the two groups.  3) Patients with the GG genotype in SNP 4 (rs4904210) were less likely to have maxillary canine impaction than those with the CC genotype and the CG genotype (OR: 0.71; 95% CI: 0.23–2.16] and (OR: 0.79; 95% CI: 0.31–2.00]. These results were not statistically significant (P > 0.05) as well.  4)For the PAX9 rs12881240) showed that 21 of 53 males and 24 of 68 females in the case group harbored the CT genotype, whereas only 4 males and 2 females harbored the TT genotype.  5)For the PAX9 (rs4904210), 26 of 53 males and 36 of 68 females in the case group had a CG genotype, whereas 13 males and 13 females had the GG genotype. | Νo statistically significant association between PAX9 genotype and maxillary canine impaction was found, although the variants rs12881240 and rs4904210 showed a greater risk for maxillary canine impaction.  There were no significant differences in gender and maxillary canine impaction. The study also suggests a potential role for PAX9 in tooth growth and development. |
| Olsson et al. (2020) [42] | 1) Bone was removed around the surfaces of impacted teeth leading to their extraction.  2) The gathered bone was placed in RNA solution and refrigerated immediately.  3) RNA was isolated using the mirVana miRNA isolation kit.  4) A StepOnePlus Real Time Polymerase Chain Reaction (PCR) system was used to run both the reverse transcription reaction and the quantification of RNA (gene expression) in duplicate.  5) The TaqMan Gene Expression Assay primers and probes were used for the real time qPCR.  6) The relative amounts of mRNA expression were calculated by the 2-Δ Cycle Threshold (2-ΔΔCT) method.  7) Prism GraphPad (GraphPad Software, Inc) was used for statistical analysis. | MSX1  RUNX2  BMP2 | No rs variant was mentioned. | 1) During the comparison of RUNX2, BMP2, and MSX1 gene expressions between maxilla and mandible the study showed that there were no significant differences.  2) RUNX2, BMP2, and MSX1 expressions according to Winter’s third molar position also showed no significant differences.  3) The expressions of RUNX2, BMP2, and MSX1 according to the Pell and Gregory classification for both maxillary and mandibular third molars showed that there was no significant difference again.  4) The analysis according to the Pell and Gregory classification stratified by the dental arch showed: a significance difference for MSX1 expression in the maxilla, in which the occlusal plane of the impacted third molar was at the same level as the occlusal plane of the second molar, and showed that there was increased expression compared with that of the tooth impacted below the cervical margin of the second molar (*p* < 0.029). | The DNA variations (were not shown) of the MSX1 gene lead to a different expression of it among the maxillary third molars, signalizing its importance in developmental alterations in their position and broadly in their impaction. |
| Adeyemo et al. (2021) [44] | 1) DNA was extracted from saliva samples.  2) After the completion of the PCR process, the plates were read using the 7900HT fast real-time PCR system controlled by SDS software version 2.4. All the files with samples and markers were imported into Progeny version 7.6.04 from Progeny software LLC USA to check for Mendelian errors and discrepancies in each file.  3) Case–control analyses were conducted to determine association using PLINK.  4) The height of the cases and controls was analyzed and compared using independent *T*-test. Alpha of *p*< 0.05 was set to determine statistical significance. | WNT9  AXIN2  MSX1  PAX9  LINC01396 | rs735510  rs1042484  rs115200552  rs12532  rs2295218  rs731120  rs6504591  rs7224837  rs11867417  rs3923086  rs2240308 | 1) No difference was found in allele frequency between cases and controls for 10 of the 11 SNPs.  2) Rs6504591 the *p* value was near significance (*p*= 0.07) with an odd ratio of 2.131.  3) Subjects with lower third molar impactions were significantly shorter than those who have fully erupted third molars. | Individuals with third molar impaction tend to have T allele at the locus, suggesting that the T allele at the locus may increase the risk of having an impacted third molar. The rs6504591 G/T variation on human chromosome 17 (WNT9B gene) appears to increase risk by two folds for impaction. |
| Ahmadi et al. (2021) [46] | 1) Clinical oral examinations, X-ray panoramic, and CBCT images investigation were undertaken to diagnose bony-impacted teeth  2) A separate survey containing information on sex, age, blood group type, and the number of impacted teeth (if there are) was used as a record for each participant.  3) A Chi-Square test was applied to determine the correlation between ABO blood groups and the prevalence of impacted teeth. All the statistical analyses were performed using Statistical Package for the Social Sciences (SPSS version, 23). | ABO and RH blood groups. | N/A | 1) 205 participants (64%) did not have any impacted ones, 26 (8%) had one, 43 (13%) had two, 5 (1%) had three, and 41 (12%) had four impacted third molars.  2)Women had a higher prevalence of the impacted third molar, as 73 (39%) of the female participants and 42 (30%) of the male participants had at least one impacted third molar.  3) Our data showed that the subjects with no impacted third molar in the blood groups A, B, AB, and O were distributed as 51 (24%), 51 (24%), 51 (24%), and 52 (25%). In addition, among subjects with different ABO blood groups, 11 (26%) participants with blood group A, 10 (24%) participants with blood group B, 8 (19%) participants with blood group AB, and 12 (29%) participants with blood group O had all third molars impacted.  4) Participants with negative RH, 112 (54%) of them had no impacted third molar and this figure was 93 (45%) for individuals with positive RH. | The evaluation of the relationship between the blood group and the impacted third molar revealed that blood groups have no association with the impacted third molars. |
| Alotaibi et al. (2021) [35] | 1) Multivariate GWAS.  2)R v3.4.1 statistical analysis. | MSX1  MSX2  ARNT2  NTN1  TRPC4  WNT2B | rs4868444  rs140220410  rs9913511  rs2251904  rs1838002 | 1) Increased rate in unaffected relatives for impaction (80.49% vs. 19.51%, *p* = 0.01).  2) MSX2 variant rs4868444 is necessary for proper craniofacial morphogenesis and odontogenesis.  3) ARNT2 variant rs140220410 regulates developmental genes and is expressed in molars and incisors.  4) NTN1 variant rs9913511 affects development of the craniofacial region in animal models and is critical for palatal fusion.  5) TRPC4 variant rs2251904 is involved in neurotransmitter release and cell proliferation and is highly expressed highly expressed in the rat dental follicle.  6) WNT2B variant rs1838002 is included in differentiation and proliferation of cementoblasts and odontoblasts. | This study emphasizes the importance of multiple genes in structural dental anomalies and specifically of the MSX2, ARNT2, NTN1, TRPC4, WNT2B genes in the craniofacial morphogenesis and odontogenesis. There isn’t enough evidence on whether these genes cause tooth impaction. |
| Trybek et al. (2021) [36] | 1) Biological material was collected using epithelial mouth swabs.  2) Genotyping was performed with polymerase chain reaction (PCR) derived from Real-Time PCR techniques using TaqMan probes.  3) Statistical analysis with the Chi-square test. | MSX1  MSX1 | rs12532  rs8670 | 1) 204 patients  1 impacted tooth -> N=11  2 impacted teeth -> N=55  3 impacted teeth -> N= 28  4 impacted teeth -> N=72  5 impacted teeth -> N=3  6 impacted teeth -> N=1  7 impacted teeth -> N=1  2) The most frequently impacted tooth was the molar, especially the lower third molars.  75.5% related to tooth #38, and 70.6% to tooth #48,  54.4% (tooth #28) and 52.2% (tooth #18)  5.4% of the #23 and 2.9% of tooth #13.  3) The analysis of rs8670 and rs12532 in the MSX1 gene showed no statistically significant differences in the frequency of genotypes (*p =* 0.487 and *p* = 0.925, respectively) and in the frequency of alleles (*p* = 0.228 and p = 0.975, respectively).  4) Three haplotypes of the MSX1 gene were identified (rs8670; rs12532): (C; A), (C; G), (T; A) with a total frequency of 100%.  5) Statistically significant differences in the frequency of genotypes described for rs12532 in the MSX1 gene were found: the occurrence of the AA genotype was more distinct in people from the NIT>MED subgroup who had more than three teeth impacted, compared to the NIT≤MED subgroup with a smaller number of impacted teeth (66.2% vs. 46.8%, *p* = 0.026). | In summary, this study underlines the higher frequency of the A allele at rs12532 of the MSX1 gene in the subgroup of patients with a bigger number of impacted teeth, compared to the subgroup in which only three or fewer impacted teeth were found (f. A 0.8 vs. 0.68, *p* = 0.009).  The rs8670 of the MSX1 gene didn’t show any significant difference in its frequency between the subgroups. |
| Bozgeyik et al. (2022) [41] | 1) Radiographic examination via panoramic radiograph.  2) After extraction the tooth follicle was removed intact without damaging it.  3) For the purification of total RNA from tissue samples of patients, RNeasy Mini Kit (Qiagen) was used. Following transfer of tissue lysate, on column DNase digestion was performed to eliminate genomic DNA.  4) QuantiTect Reverse Transiptase Kit (Qiagen) was used for the cDNA synthesis of RNA samples.  5) Real-time PCR and The QuantiTect SYBR Green PCR Kit (Qiagen) were used to determine the quantitative expression of T-UCRs.  6) For the statistical analysis GraphPad Prism 9 and SPSS 28 software were used. | T-UCRs  1) uc.38  2) uc.112  3) uc.338 | No rs variant was mentioned. | 1) The study found that uc.38, uc.112 and uc.338 were distinctly expressed in the follicle samples compared to the healthy gingival tissue, and their expression was higher in the follicle tissue samples.  2) The uc.38 expression level was approximately 1.55-fold in the follicle sample.  3)The uc.112 expression level was approximately 1.98-fold higher in the follicle sample.  4)The uc.338 expression level was approximately 7.71-fold in the follicle sample. | This study marks the significant presence of these T-UCRs: uc.38, uc.112, uc.338 in the dental follicle of impacted mandibular third molars. This discovery shows us that these T-UCRS are responsible for the molecular changes in the tissues surrounding the impacted teeth. |
| Ege et al. (2022) [28] | 1) The appropriate sample size was analyzed with the help of G*Power.  2) Panoramic radiographs of patients were obtained with the Planmeca, ProMax (Planmeca Oy, Helsinki, Finland) instrument prior to surgical procedure. The panoramic images obtained were transferred to the digital environment via Planmeca Romexis (Planmeca Oy, Helsinki, Finland) software, and the pericoronal intervals of impacted mandibular third molars (IMTM) were measured on these images based on the widest points of the follicle with IMTM.  3) After the extraction was completed, the extraction socket was irrigated with sterile saline solution. After an effective hemostasis was achieved, the extraction socket was curetted, and the dental follicle was made visible and removed intact without damaging.  4) Isolation of total RNA from dental follicle and healthy gingival tissue samples was carried out with the aid of GeneJET RNA Purification Kit (Thermo Scientific, USA). Briefly, 30 mg of tissue was weighed and homogenized by using TissueLyser LT (Qiagen, Germany) in lysis buffer containing β-mercaptoethanol. Subsequently, Proteinase K was added to the homogenized solution and left for incubation of 10 min at ambient temperature.  5) The RNA containing solution was transferred to RNA purification columns and columns were washed with the washing solutions. Total RNA was eluted using supplied elution buffer, and purity and concentration of the obtained RNAs were measured by the aid of NanoDrop 2000 instrument. RNA concentrations adjusted to 500 ng and stored at − 80 °C until further steps.  6) RevertAid First-Strand cDNA Synthesis Kit (Thermo Scientific, USA) was used for the synthesis of cDNA from RNA samples.  7) Gene-specific DNA oligonucleotides were designed to determine the expression levels of TP73-AS1, NORAD, MEG3, and MALAT1 lncRNA genes in the dental follicle tissue samples and GAPDH (glyceraldehyde 3-phosphate dehydrogenase) reference control gene was used for the normalization of the expression levels of these genes.  8) Forward and Reverse primers at a concentration of 0.3 µM, 2 µl of cDNA, 10 µl of 2X SYBR® Green PCR Master Mix (Thermo Scientific, USA), and molecular biology-grade nuclease-free water up to a total volume of 20 µl was prepared. Then, prepared reactions were subjected to thermal conditions for 10 min at 95°C, 30 s at 95°C, 30 s at 60°C, and 30 s at 72°C for 40 cycles using Rotor Gene Real-Time PCR instrument (Qiagen, Germany  9) GraphPad Prism (v6.02) and SPSS (v16.0) package programs were used for statistical analysis. To determine the expression levels, the relative gene expression results were calculated with the formula 2^−ΔCt^ (ΔCt = Ct_target gene_ − Ct_reference gene_) | LncRNAs:  MALAT1  TP73-AS1  NORAD  MEG3 |  | 1) The gene expression of MEG3 increased about 10-fold in DF tissues of impacted third molars compared to healthy gingival tissues (*p* < 0.0001).  2) NORAD expression was found to be upregulated 4.2-fold (*p* = 0.0002) in DF tissues.  3) Expression level of MALAT1 was found to be decreased 1.24-fold (*p* = 0.584) and TP73-AS1 increased 2.6-fold (*p* = 0.093) in DF tissues compared to healthy gingival tissues. | Different lncRNA expressions in dental follicles may be effective in processes such as osteogenic differentiation, DNA damage, and the transformation into odontogenic pathology. In particular, the expression level of MEG3 and NORAD lncRNA molecules may guide clinicians in the evaluation of asymptomatic impacted third molars (ITM) dental follicles that cannot be determined radiologically and during extraction of these teeth for prophylactic purposes. |
| Alassaf et al. (2023) [47] | 1) Two investigators screened the existing radiographs, contacted the patients who met the inclusion criteria to obtain the blood group as registered in the patient's health card by the Ministry of Health.  2) Data was coded and analysis was done using Statistical Package for the Social Sciences (SPSS version 23). A descriptive analysis of the variables as percentage and frequency for qualitative data and mean and standard deviation for qualitative variables was conducted. Then, in the comparison process between groups, chi-squared tests were used with a significance level set at a *p*-value of ≤0.05. | ABO and Rh blood groups. | N/A | No statistically significant association was found among the blood groups. | There is no association between blood type and wisdom tooth impaction. However, the study was limited in diversity. |
| Trybek et al. (2023) [20] | 1) DNA was extracted from venous blood.  2) Genotyping was conducted with real-time PCR.  3) All analyses were performed using STATISTICA 13. | PAX9  MSX1  MSX1  AXIN2  AXIN2  AXIN2  IRF6  IRF6  IRF6  IRF6 | rs4904210  rs8670  rs12532  rs7591  rs2240308  rs4904210  rs642961  rs861019  rs4904210  rs658860 | 1) The PAX9 rs4904210, AXIN2 rs7591, AXIN2 rs4904210, IRF6 rs642961, IRF6 rs861019, IRF6 rs4904210, and IRF6 rs658860 genotype did not differ in the expression of impacted teeth.  2) The AXIN2 rs2240308, MSX1 rs12532 and MSX1 rs8670 genotypes have a particularly important role in tooth impaction both in maxilla and mandible together and in maxilla and mandible separately. | This study highlights the importance of the AXIN2 rs2240308, MSX1 rs12532 and MSX1 rs8670 genotype specifically in tooth impaction and in general of the PAX9 rs4904210, AXIN2 rs7591, AXIN2 rs4904210,  IRF6 rs642961,  IRF6 rs861019,  IRF6 rs4904210, and IRF6 rs658860 genotype. |
| Almalki et al. (2024) [45] | 1) The sample size was determined using the formula n = z²pq/d², considering p as 25% and allowable error 5%. The calculation yielded a sample size of 288, which was rounded off to 300.  2) Purposive sampling was used as sampling method. Data was collected when patients came for initial screening and participants were provided with comprehensive information about the study’s objectives and procedures. Informed consent was obtained from all participants, and certified records such as driving licenses and sehaty applications provided by the government based on the records that provided data of blood groups of the participating subjects were used to record the blood groups.  3) Dental caries was assessed using the WHO modified criteria (1986) on the DMF-Index (Decayed, Missing, Filled Teeth) and the DMFS (Decayed, Missing, Filled Surfaces) index by Henry T. Klien, Carrole E. Palmar, and Knutson J.W. (1938). The presence or absence of gingivitis was determined using the Gingival Index (GI) by Loe and Silness (1963).  4) One-way Analysis of Variance (ANOVA) was used to assess the impact of blood groups on DMFT and DMFS scores, and comparative analyses between groups were conducted using chi-square analysis and Spearman correlation for qualitative data. | ABO Blood groups. | N/A | Impacted molars:  Blood Groups: A - B – AB - O  Present N (%): 26(25) - 14(13.5) - 39(37.5) - 41(21)  Absent N (%): 53(27) - 38(19.4) - 41(21) - 64(32.6) | Individuals with AB blood group are more prone to impaction of molars. |

**Table A4:** Studies excluded with main reason for exclusion.

| Author (et al.) | Year | Title | Inclusion – Exclusion Reason |
| --- | --- | --- | --- |
| Abdoli | 2010 | Reduction of dental filling metallic artifacts in CT-based attenuation correction of PET data using weighted virtual sinograms optimized by a genetic algorithm | wrong outcome |
| Adler | 2011 | Survival and recovery of DNA from ancient teeth and bones | wrong outcome |
| AF | 1984 | Problems of impacted teeth retrospective study of 1620 cases | wrong study design |
| Aggarwal | 2021 | The bridge connecting sella turcica and palatally impacted canine. | wrong outcome |
| Aktan | 2010 | The incidence of canine transmigration and tooth impaction in a Turkish subpopulation | wrong outcome |
| Al-Abdallah | 2018 | What factors affect the severity of permanent tooth impaction? | wrong outcome |
| Ali | 2014 | Association between sella turcica bridging and palatal canine impaction. | wrong outcome |
| Ali | 2023 | Inverted Impacted Teeth: Rare Case Series of 13 Cases. | wrong study design |
| Alling | 1979 | Impacted canine teeth | wrong outcome |
| Alling | 1993 | Indications for management of impacted teeth | wrong outcome |
| Almarhoumi | 2022 | Frequency and pattern of impacted Canines in Al-Madinah, Saudi Arabia: A cross-sectional radiographic study. | wrong outcome |
| Al-Noaman | 2022 | A CORRELATION BETWEEN RADIOGRAPHICAL ASSESSMENT AND POST-OPERATIVE COMPLICATIONS OF IMPACTED TEETH | wrong outcome |
| Alotaibi | 2019 | Genetic Analyses of Dental Anomalies and Dental Caries in Multiethnic Populations | wrong outcome |
| Alqerban | 2016 | Early prediction of maxillary canine impaction. | wrong outcome |
| Alqerban | 2015 | Radiographic predictors for maxillary canine impaction. | wrong outcome |
| Altaweel | 2022 | A novel therapeutic approach for controlling complications associated with impacted mandibular third molar removal | wrong outcome |
| Amira | 2024 | Modeling and multi-objective optimization of the milling process for AISI 1060 steel | wrong outcome |
| Andreoni F | 2015 | Missense mutations in EDA and EDAR genes cause dominant syndromic tooth agenesis | medical condition |
| Antunes | 2022 | Genetic polymorphisms in TNF-Î± as a potential biomarker for oral health-related quality of life in children | wrong outcome |
| Aslan | 2015 | Clinical consideration and management of impacted maxillary canine teeth | wrong outcome |
| Athanasiou | 2024 | Palatal canine impaction is associated with craniofacial shape in humans | wrong outcome |
| Aucott | 2021 | Medical biotechnology as a paradigm for forest restoration and introduction of the transgenic American chestnut | wrong outcome |
| Avsever | 2017 | Multicentre evaluation of impacted and transmigrated canines: a retrospective study | wrong outcome |
| Aydin | 2004 | Incidence of canine impaction and transmigration in a patient population. | wrong outcome |
| Azaz | 1977 | Aging of tissues of the roots of nonfunctional human teeth (impacted canines) | wrong outcome |
| Azaz | 1974 | Correlation between age and thickness of cementum in impacted teeth | wrong outcome |
| Babacan | 2008 | Identical unerupted maxillary incisors in monozygotic twins | wrong study design |
| Bakhsh | 2016 | Simplified treatment mechanics with a miniscrew for a case of canine impaction. | wrong outcome |
| Balaji | 2020 | Impacted wisdom tooth in the floor of the orbit | wrong outcome |
| Balasubramanian | 2023 | Design and Optimization of Interior Permanent Magnet (IPM) Motor for Electric Vehicle Applications | wrong outcome |
| Basdra | 2015 | Congenital tooth anomalies and malocclusions: a genetic link? | medical condition |
| Baumann | 2017 | Stable Isotopes and Oral Tori in Greenlandic Norse and Inuit | outcome |
| Baumgardner | 2020 | Mandibular feed impaction resulting in fistulous tract development in a kunekune sow (Sus scrofa domesticus) | wrong outcome |
| Bayar | 2008 | Multiple impacted teeth: report of 3 cases | wrong study design |
| Bazurto | 2015 | Aminoimidazole carboxamide ribotide exerts opposing effects on thiamine synthesis in Salmonella enterica | wrong outcome |
| Becker | 2010 | Extreme tooth impaction and its resolution | wrong outcome |
| Becker | 2015 | Impacted teeth and the six incarnations of resorption | wrong outcome |
| Becker | 1983 | Periodontal status following the alignment of palatally impacted canine teeth | wrong outcome |
| Becker | 2015 | Etiology of maxillary canine impaction: a review | wrong study design |
| Becker | 2016 | The etiology of palatal displacement of maxillary canines | wrong study design |
| Becker | 1997 | Palatal displacement of canine is genetic and related to congenital absence of teeth | wrong outcome |
| Beech R | 1994 | The resource implications and service outcomes of genetic services in the context of DNA technology | wrong outcome |
| Benderlioglu | 2010 | Fluctuating Asymmetry and Steroid Hormones: A Review | wrong study design |
| Bharathi | 2022 | Association of vertical growth pattern with canine impactions in Dravidian subjects. | wrong outcome |
| Bianchi | 1991 | Primary impaction of primary teeth: a review and report of three cases. | wrong study design |
| Bihlmeyer | 2014 | Genetic diversity is a predictor of mortality in humans | wrong outcome |
| Bitencourt | 2018 | Impact of genetic variations in the WNT family members and RUNX2 on dental and skeletal maturation: a cross-sectional study | wrong outcome |
| Blin-Wakkach | 2017 | Endogenous Msx1 antisense transcript: in vivo and in vitro evidences, structure, and potential involvement in skeleton development in mammals | wrong outcome |
| Bocutog | 1997 | Coronal displacement of cementum: Correlation between age and coronal movement of cementum in impacted teeth | wrong outcome |
| Bodner | 2001 | Image accuracy of plain film radiography and computerized tomography in assessing morphological abnormality of impacted teeth | wrong study design |
| Bolar | 2023 | Sustainable thin-wall machining: holistic analysis considering the energy efficiency, productivity, and product quality | wrong outcome |
| Borah | 2024 | Development of ANN model for predicting mechanical properties of 3D printed PEEK polymer using FDM and optimization of process parameters for better mechanical properties | wrong outcome |
| Bornstein | 2010 | The use of three-dimensional reconstructions in the diagnosis of impacted teeth | wrong outcome |
| Borns-Weil | 2015 | A case-control study of compulsive wool-sucking in Siamese and Birman cats (n = 204). | wrong outcome |
| Bozzay | 2010 | Dentition anomalies within a family | wrong study design |
| Bracco | 2012 | Etiopathogenesis and classification of impacted teeth | wrong outcome |
| Brakel | 2021 | Exploring, harnessing and conserving marine genetic resources towards a sustainable seaweed aquaculture | wrong outcome |
| Brkić | 2018 | Pathological changes and immunoexpression of p63 gene in dental follicles of asymptomatic impacted lower third molars: an immunohistochemical study | wrong outcome |
| Brown | 1982 | A radiological study of the frequency and distribution of impacted teeth | wrong outcome |
| Cabanillas | 2023 | Status of the largetooth sawfish in Ecuador and Peru, and use of rostral teeth in cockfighting | wrong population |
| Cahill | 1969 | Eruption pathway formation in the presence of experimental tooth impaction in puppies | wrong population |
| Cahill | 1970 | The histology and rate of tooth eruption with and without temporary impaction in the dog | wrong population |
| Cain | 2014 | Sex-biased inbreeding effects on reproductive success and home range size of the critically endangered black rhinoceros | wrong population |
| Cakan | 2018 | The genetic basis of dental anomalies and its relation to orthodontics | wrong outcome |
| Cakan | 2019 | The genetic basis of facial skeletal characteristics and its relation with orthodontics | wrong outcome |
| Camilleri | 2013 | The genetic aetiology of ectopic maxillary canine teeth | wrong outcome |
| Cammarata | 2018 | Main genetic entities associated with supernumerary teeth | medical condition |
| Cao | 2021 | Root dilaceration in maxillary impacted canines and adjacent teeth: A retrospective analysis of the difference between buccal and palatal impaction. | wrong outcome |
| Carels | 2010 | Role of inheritability of tooth form, tooth malformation and tooth position | wrong outcome |
| Carl | 1972 | Impacted teeth in denture-bearing areas: A potential source of problems | wrong outcome |
| Carl | 1995 | Impacted teeth: prophylactic extractions or not? | wrong outcome |
| Cassetta | 2018 | Maxillary canine impaction and the association with dental and skeletal anomalies: A retrospective study | wrong outcome |
| Cassetta | 2020 | Relationship between upper lateral incisors anomalies and palatal displaced canine: a case-control retrospective study | wrong outcome |
| Casto | 1921 | Impacted deciduous teeth | wrong outcome |
| Celik | 2022 | Deep learning based detection tool for impacted mandibular third molar teeth | wrong outcome |
| Celikoglu | 2010 | Frequency of agenesis, impaction, angulation, and related pathologic changes of third molar teeth in orthodontic patients | wrong outcome |
| Celikoglu | 2010 | Investigation of transmigrated and impacted maxillary and mandibular canine teeth in an orthodontic patient population | wrong outcome |
| Chandak | 2014 | Comparative study of dentascan and radiography for radiological evaluation of impacted teeth | wrong outcome |
| Chaushu | 2005 | Patients' perception of recovery after exposure of impacted teeth: a comparison of closed-versus open-eruption techniques | wrong outcome |
| Chelliah | 2024 | Prediction of Favorability of Maxillary Canine Impaction Using Artificial Intelligence Algorithm. | wrong outcome |
| Chen | 2025 | Causal inferences on childhood obesity and dentofacial anomalies: a mendelian randomization study | medical condition |
| Choi | 1972 | Roentgenographical observation of impacted teeth | wrong outcome |
| Clark | 2024 | Exploring the journey to genomic testing and genetic services: A qualitative study of parental perspectives of children with rare conditions | wrong outcome |
| Coşarcă | 2016 | The evaluation of Ki67, p53, MCM3 and PCNA immunoexpressions at the level of the dental follicle of impacted teeth, dentigerous cysts and keratocystic odontogenic â€¦ | wrong outcome |
| Consolaro | 2019 | Impacted teeth: Their place is in the dental arch | wrong outcome |
| Cooper | 2021 | DLX6 and MSX1 from saliva samples as potential predictors of mandibular size: A cross-sectional study | wrong outcome |
| Crincoli | 2019 | Correlation Between Maxillary Canine Impaction and Facial Biotype. | wrong outcome |
| Dürwald | 2020 | Tooth Eruption Disorders - Causes and Symptoms | medical condition |
| Díaz |  | Impacted teeth (Impaction of upper central incisors in twins) | wrong study design |
| Dachi | 1961 | A survey of 3,874 routine full-mouth radiographs: II. A study of impacted teeth | wrong outcome |
| Daher | 2018 | Bcl11b/Ctip2 in Skin, Tooth, and Craniofacial System | wrong outcome |
| Dalessandri | 2018 | Impacted and transmigrant mandibular canines | wrong study design |
| Daniels | 2016 | Unmasking alpha diversity, cladogenesis and biogeographical patterning in an ancient panarthropod lineage (Onychophora: Peripatopsidae: Opisthopatus cinctipes) with the description of five novel species | wrong outcome |
| Davarpanah | 2009 | Unconventional implant placement. 2: placement of implants through impacted teeth. Three case reports. | wrong study design |
| de Souza | 2020 | Analysis of on farm conservation of sweet corn in a diversity microcenter of Zea mays L. in Southern Brazil | wrong population |
| Dekel | 2021 | Impaction of maxillary canines and its effect on the position of adjacent teeth and canine development: A cone-beam computed tomography study. | wrong outcome |
| Dereudre | 2001 | Rare earth magnets in conjunction with fixed orthodontics. An" attractive" solution for the positioning of impacted teeth | wrong outcome |
| Dern | 2023 | The impact of medieval and early modern migrations on dental nonmetric variation in Hungary | wrong outcome |
| Dhamo | 2015 | The association between WNT10A variants and dental development in patients with isolated oligodontia | wrong outcome |
| Di Donato | 1998 | The complex clinical and genetic classification of inherited ataxias. I. Dominant ataxias | wrong outcome |
| Diedrich | 2023 | Extinct Eurasian rhinoceros Coelodonta and Stephanorhinus dental pathologies and tooth change modus | wrong outcome |
| Ding | 2020 | Novel MSX1 gene variants in chinese children with non-syndromic tooth agenesis: a clinical and genetic analysis | medical condition |
| Duval | 2018 | The first direct ESR dating of a hominin tooth from Atapuerca Gran Dolina TD-6 (Spain) supports the antiquity of Homo antecessor | wrong outcome |
| Dye | 2009 | Humans are still evolving but technology will speed the process | wrong outcome |
| Eidelman | 1979 | Fatigue on Rest and associated symptoms (headache, vertigo, blurred vision, nausea, tension and irritability) due to locally asymptomatic, unerupted, impacted teeth | wrong outcome |
| Elsner | 2015 | Burial condition is the most important factor for mtDNA PCR amplification success in Palaeolithic equid remains from the Alpine foreland | wrong outcome |
| Esposito | 2008 | Impacted wisdom teeth. | wrong outcome |
| Fenoglio | 2006 | Hippocampal neuroplasticity induced by early-life stress: Functional and molecular aspects | wrong outcome |
| Ferguson | 1926 | Impacted Tooth with Infection | wrong outcome |
| Fernandez | 2019 | Genes candidatos as anomalias dentarias em pacientes portadores de maloclussion esquel anttica | wrong outcome |
| Finegan | 2019 | Widespread subcortical grey matter degeneration in primary lateral sclerosis: a multimodal imaging study with genetic profiling | wrong outcome |
| Finley | 2023 | Exploring the impact of virtual SPIKES training on genetic counselors' confidence to deliver difficult news | wrong outcome |
| Fischer | 2014 | The study of barium concentration in deciduous teeth, impacted teeth, and facial bones of Polish residents | wrong outcome |
| Fleischmannova | 2010 | Formation of the tooth-bone interface | wrong study design |
| Fonseca | 2019 | Single-Nucleotide Polymorphisms in MMP3, TIMP1, and MTR Genes are associated with delayed deciduous tooth eruption | wrong outcome |
| Forsberg | 1988 | Tooth size, spacing, and crowding in relation to eruption or impaction of third molars | wrong outcome |
| Frazier-Bowers | 2015 | An interview with Sylvia Frazier-Bowers. | wrong outcome |
| Frazier-Bowers | 2016 | The etiology of eruption disorders - further evidence of a 'genetic paradigm' | wrong outcome |
| Friedrich | 2003 | The influence of wisdom tooth impaction on root formation | wrong outcome |
| Gamarra | 2022 | Identifying biological affinities of Holocene northern Iberian populations through the inner structures of the upper first molars | wrong outcome |
| Gang | 1991 | (Impacted tooth) | wrong outcome |
| Gao | 2024 | The relationship of impacted maxillary canines and maxillary skeletal and dental size and shape: A Cone Beam Computed Tomography Study | wrong outcome |
| Garib | 2009 | Increased occurrence of dental anomalies associated with second-premolar agenesis | medical condition |
| Gasilin | 2023 | Geographic variations in the shape of the upper toothrow of the artcic fox vulpes lagopus l. 1758: effects of asymmetry and allometry | wrong population |
| Gasimova | 2014 | Contemporary approach to diagnosis and treatment of impacted teeth | wrong outcome |
| Gerber | 2021 | Odontogenesis-related candidate genes involved in variations of permanent teeth size | wrong outcome |
| Ghadimi | 2017 | Associations among sella turcica bridging, atlas arcuate foramen (ponticulus posticus) development, atlas posterior arch deficiency, and the occurrence of palatally displaced canine impaction. | wrong outcome |
| Giovannetti | 2019 | Small RNAs and tooth development: The role of microRNAs in tooth agenesis and impaction | wrong study design |
| Giuliana | 1995 | Cementum growth in impacted teeth. | wrong outcome |
| Gomes | 2010 | Digging deeper into East African human Y chromosome lineages. | wrong outcome |
| González | 2024 | Arsenic inorganic exposure, metabolism, genetic biomarkers and its impact on human health: A mini-review | wrong study design |
| Gorski | 2006 | Postnatal environment overrides genetic and prenatal factors influencing offspring obesity and insulin resistance | wrong outcome |
| Govindasamy | 2022 | Emerging trends of nanotechnology and genetic engineering in cyanobacteria to optimize production for future applications | wrong outcome |
| GU | 2001 | Clinical application and evaluation of spiral ct in location with impacted teeth | wrong outcome |
| Guerroudj | 2017 | Performance analysis of Vernier slotted doubly salient permanent magnet generator for wind power | wrong outcome |
| Gunter | 1942 | Concerning impacted teeth | wrong outcome |
| Gupta | 2019 | Role of dentist in genetic counseling: A critical appraisal of the current practices and future requirements in Indian scenario | wrong outcome |
| Haidar | 1986 | The incidence of impacted wisdom teeth in a Saudi community | wrong outcome |
| Haidry | 2021 | Histopathologic changes in dental follicle associated with radiographically normal impacted lower third molars | wrong outcome |
| Harada | 2021 | Age-related changes in the effect of rapid maxillary expansion on the position of labially impacted maxillary canines: A case-control study. | wrong outcome |
| Haralur | 2017 | Incidence of impacted maxillary canine teeth in Saudi Arabian subpopulation at central Saudi Arabian region | wrong outcome |
| Harijadi | 2010 | Early removal of odontoma resulting in spontaneous eruption of the impacted teeth | wrong outcome |
| Harris | 1920 | Is an impacted tooth per se an indication for operation? | wrong outcome |
| Hart | 2002 | Genetic testing considerations for oral medicine | wrong outcome |
| Harte | 2013 | Triplets with bilateral palatally displaced canines in association with third molar agenesis: an example of biologically related dental anomaly patterns? | wrong study design |
| He | 2015 | Advances and overview of the study on paleo-earthquake events: A review of seismites | wrong study design |
| He | 2024 | Optimization design of gear with lead modification considering the effect of twist error | wrong outcome |
| Henry | 1936 | molar tooth in man based on measurements obtained from radiographs, with special reference to the problem of predicting cases of ultimate impaction of the tooth | wrong outcome |
| Hersberger | 2023 | Genetic and environmental impact on mandibular growth in mono- and dizygotic twins during adolescence: A retrospective cohort study | wrong outcome |
| Holmes | 1910 | Can impacted teeth cause moral delinquency? | wrong outcome |
| Holz | 2018 | Permanent canine eruption into the alveolar cleft region after secondary alveolar bone grafting: Are there prediction factors for impaction?. | wrong outcome |
| Hou | 2010 | Investigation of impacted permanent teeth except the third molar in Chinese patients through an X-ray study | wrong outcome |
| Hou | 2023 | Novel STAT1 mutation in a paediatric case of chronic mucocutaneous candidiasis complicated by primary hypothyroidism: clinical presentation, genetic analysis and prognostic implications | wrong study design |
| Howard | 1978 | Impacted tooth position: unexpected improvements | wrong outcome |
| Huang | 2021 | Design of gerotor pump and influence on oil supply system for hybrid transmission | wrong outcome |
| Hunt | 1961 | Malocclusion and civilization | wrong outcome |
| Hussein | 2022 | The association of polymorphisms in BMP2/MYO1H and skeletal Class II div.1 and mandibular dimensions. A preliminary report | wrong study design |
| Hylander | 2013 | Effects of coffee management on deforestation rates and forest integrity | wrong outcome |
| Ishida | 2011 | The long-term consequences of hybridization between the two Daphnia species, D galeata and D dentifera, in mature habitats | wrong study design |
| Jacobs | 1986 | Bonding characteristics of impacted versus erupted permanent teeth | wrong outcome |
| Jagiellonian University | 2015 | Closed window technique versus open window technique in management of palatally impacted canines. a randomized clinical trial | wrong outcome |
| Jain | 2020 | Permanent mandibular canine(s) impaction: expansion of our understanding | wrong outcome |
| Janečková | 2018 | Metalloproteinases are involved in the regulation of prenatal tooth morphogenesis | wrong outcome |
| Jena | 2010 | The distribution of individual tooth impaction in general dental patients of Northern India. | wrong outcome |
| Jeong | 2015 | Food impaction and periodontal/peri-implant tissue conditions in relation to the embrasure dimensions between implant supported fixed dental prostheses and adjacent teeth: A cross-sectional study | wrong outcome |
| Johnson | 2010 | Factors affecting the alkaline cooking performance of selected corn and sorghum hybrids | wrong outcome |
| Joshi | 2011 | Variations in impacted mandibular permanent molars: Report of three rare cases. | wrong study design |
| Joyce | 2014 | Bilaterally symmetrical multiple impacted permanent teeth in a nonsyndromic patient: A rare finding | wrong outcome |
| Jurado-Angulo | 2025 | Historical demographic determinants complement climate model predictions of co-occurring cryptic species | wrong outcome |
| Kamak | 2011 | Incidence and effects of genetic factors on canine impaction. | wrong outcome |
| Kamberos | 1990 | Multiple impaction teeth. Clinical and radiological study in patients with three or more impacted teeth | wrong outcome |
| Kamoun | 2012 | Genetic collagen disorders and the impact on craniofacial development | wrong outcome |
| Kantaputra | 2017 | Mutations in LRP6 highlight the role of WNT signaling in oral exostoses and dental anomalies | wrong outcome |
| Kaplan | 2024 | Determination of the frequency of impacted teeth in individuals in Tekirdag region | wrong outcome |
| Katalinić | 2012 | Diagnostics and therapy of maxillary impacted canine | wrong outcome |
| Kawame | 2001 | Teeth, impacted | wrong outcome |
| Kawana | 1990 | A pathological and histochemical study of capsules of impacted teeth with special reference to keratin immunohistochemistry in the lining epithelium | wrong outcome |
| Keane | 2005 | Pollution and genetic structure of North American populations of the common dandelion (Taraxacum officinale). | wrong outcome |
| Kelly | 2022 | Persistence in a changing world: Bison and horse dietary niche, body size, and relative abundance in late pleistocene beringia | wrong population |
| Kerdelhué | 2009 | Quaternary history and contemporary patterns in a currently expanding species | wrong outcome |
| Kessler | 2021 | Neurofilament light chain is a cerebrospinal fluid biomarker in hereditary spastic paraplegia. | wrong outcome |
| Khacho | 2017 | Mitochondrial dysfunction underlies cognitive defects as a result of neural stem cell depletion and impaired neurogenesis | wrong outcome |
| Khan | 2021 | Pathologies associated with impacted maxillary canine tooth in patients presenting at a tertiary care hospital | wrong outcome |
| Khidas | 2013 | Morphological divergence of continental and island populations of Canada lynx | wrong outcome |
| Khoury | 2016 | Impacted teeth: What should we know | wrong outcome |
| Kilby | 2021 | The role of next-generation sequencing in the investigation of ultrasound-identified fetal structural anomalies | wrong outcome |
| Kim | 2003 | Comparative Ki-67 expression and apoptosis in the odontogenic keratocyst associated with or without an impacted tooth in addition to unilocular and multilocular â€¦ | wrong outcome |
| Koshak | 2022 | Are facial, maxillary arch and incisor dimensions related in patients with a unilaterally impacted palatal canine? A prospective investigation | wrong outcome |
| Kotsomitis | 1997 | Inherited dental anomalies and abnormalities | medical condition |
| Krasny | 2020 | Alternative methods of repositioning impacted maxillary canines in the dental arch. En Bloc Autotransplantation of a Tooth | wrong outcome |
| Kroll | 2022 | Mobility and land use in the Greater Khorasan Civilization: Isotopic approaches (87Sr/86Sr, δ18O) on human populations from southern Central Asia | wrong outcome |
| Kshetrapal | 2011 | New attachment for bonding impacted teeth in closed eruption approach | wrong outcome |
| Kurol | 2006 | Impacted and ankylosed teeth: why, when, and how to intervene | wrong outcome |
| Kurosaka | 2022 | Development of dentition: From initiation to occlusion and related diseases | wrong study design |
| Lézot | 2018 | Cross-talk between Msx/Dlx homeobox genes and vitamin D during tooth mineralization | wrong study design |
| Lalos | 2007 | Legislated right for donor-insemination children to know their genetic origin: a study of parental thinking. | wrong outcome |
| Lambot | 2016 | Towards optical in vivo electrophysiology | wrong population |
| Laport | 2022 | Environmental niche and demographic modeling of american chestnut near its southwestern range limit | wrong outcome |
| Lear | 2023 | Growth and morphology of critically endangered green sawfish Pristis zijsron in globally important nursery habitats | wrong population |
| Lechien | 1995 | Should we or should we not extract impacted teeth? | wrong outcome |
| Lempesi | 2014 | A comparison of apical root resorption after orthodontic treatment with surgical exposure and traction of maxillary impacted canines versus that without impactions. | wrong outcome |
| Leonardi | 2003 | Palatally displaced canine anomaly in monozygotic twins | wrong study design |
| Levine | 2002 | The Book of Agreement:10 Essential Elements for Getting the Results You Want | wrong outcome |
| Li | 2009 | Experience introduction of induced eruption on multiple adjacent impacted teeth in anterior maxillary bone | wrong outcome |
| Li | 2016 | Genome-Wide Association study reveals the genetic basis of stalk cell wall components in maize | wrong outcome |
| Li | 2021 | PITX2 expression and Neanderthal introgression in HS3ST3A1 contribute to variation in tooth dimensions in modern humans | wrong outcome |
| Li | 2023 | NetMUG: a novel network-guided multi-view clustering workflow for dissecting genetic and facial heterogeneity | wrong outcome |
| Liao | 2017 | The Sirt6 gene: Does it play a role in tooth development? | wrong population |
| Lifton | 1938 | Impacted and unerupted teeth and their recovery | wrong outcome |
| Lin | 2015 | The genetic and environmental contributions to variation in the permanent dental arch form: a twin study | wrong study design |
| Lin | 2018 | The epigenetic regulation in tooth development and regeneration | wrong study design |
| Logan | 1921 | Should all pulpless and impacted teeth be removed? | wrong outcome |
| Lucentini | 2023 | Long Eared Owls (Asio otus Linnaeus, 1758) as field-assistants in an integrative taxonomy survey of a Peculiar Microtus savii (Rodentia, Cricetidae) population | wrong population |
| Lupinetti | 2022 | Non-syndromic hypodontia of maxillary lateral incisors and its association with other dental anomalies | medical condition |
| Macalino | 2023 | A study of disparities in access to genetic care pre- and post-pandemic | wrong outcome |
| Maguolo | 2020 | Diagnosis, genetic characterization and clinical follow up of mitochondrial fatty acid oxidation disorders in the new era of expanded newborn screening: A single centre experience | wrong outcome |
| Mahabob | 2020 | Transmigration of mandibular cuspids: Review of literature. | wrong study design |
| Mallawaarachchi | 2025 | Enhancing diagnostic outcomes in kidney genetic disorders: the KidGen national kidney genomics study protocol | wrong outcome |
| Manoj | 2015 | Rare patterns of impacted mandibular teeth: A case series. | wrong study design |
| Martín-Francés | 2020 | Ectopic maxillary third molar in Early Pleistocene Homo antecessor from Atapuerca-Gran Dolina site (Burgos, Spain) | wrong outcome |
| Martis | 1978 | Extractions of impacted mandibular wisdom teeth in the presence of acute infection | wrong outcome |
| Mcdaniel | 1935 | Dental instrument for removing impacted teeth | wrong outcome |
| Mead | 1930 | Incidence of impacted teeth | wrong outcome |
| Mercuri | 1980 | Multiple impacted and supernumerary teeth in sisters | wrong study design |
| Mesotten | 2005 | Bilaterally impacted maxillary canines and multiple missing teeth: a challenging adult case | wrong study design |
| Michie | 1997 | Non directiveness in genetic counseling: an empirical study | wrong outcome |
| Michon | 2011 | Tooth evolution and dental defects: from genetic regulation network to micro-RNA fine-tuning | wrong outcome |
| Miller | 2014 | Exploration of a rare population of Chinese chestnut in North America: stand dynamics, health and genetic relationships | wrong outcome |
| Mitra | 2022 | Epidemiology of major entero-pathogenic viruses and genetic characterization of Group A rotaviruses among children (≤5 years) with acute gastroenteritis in eastern India, 2018-2020 | wrong outcome |
| Mohammad | 2020 | Correlation between skeletal development and maxillary canine eruption. | wrong outcome |
| Montelius | 1932 | Impacted teeth: a comparative study of Chinese and Caucasian dentitions | wrong outcome |
| Mu | 2021 | Multiobjective ease-off optimization of high-speed spiral bevel gear for loaded meshing performance | wrong outcome |
| Muthukumar | 2018 | Sex determination of an individual by studying the mandibular canine index | wrong outcome |
| Nachat | 2024 | Etiologia, diagnostico e abordagem terapeutica de caninos inclusos maxilares no tratamento ortodontico | wrong outcome |
| Nagahara | 1989 | Etiological study of relationship between impacted permanent teeth and malocclusion | wrong outcome |
| Nagpal | 2017 | Palatal and labially impacted maxillary canine-associated dental anomalies: a comparative study | medical condition |
| Nakamura | 1992 | Rosette formation of impacted molar teeth in mucopolysaccharidoses and related disorders | wrong outcome |
| Narang | 2016 | Dermatoglyphics (finger prints) as predilection marker for impacted teeth: A randomized blind trial | wrong outcome |
| Nataliia | 2020 | A new look at retented and impacted teeth | wrong outcome |
| Natarajan | 2024 | Geometric morphometric shape analysis of mandibular post-canine dentition | wrong outcome |
| Nino-Rosales | 2017 | Genes underlying familial hypodontia: a review and discussion of the role of dental hygienists in future research | wrong study design |
| Nitzan | 1986 | The effect of aging on tooth morphology: a study on impacted teeth | wrong outcome |
| Nuvvula | 2011 | Atypical presentation of bilateral supplemental maxillary central incisors with unusual talon cusp. | wrong outcome |
| Oikarinen | 1990 | Similarly impacted second and third maxillary and mandibular molars in a pair of monozygotic twins | wrong study design |
| Okada | 2002 | Intraosseous tooth migration of impacted mandibular premolar: computed tomography observation of 2 cases of migration into the mandibular neck and the coronoid process | wrong study design |
| Olin | 1989 | Classification of impacted teeth | wrong outcome |
| Oppenheim | 2007 | Salivary proteome and its genetic polymorphisms | wrong outcome |
| Orr | 1995 | We could do with a few less holes in the head Michigan's miserable weather can plague susceptible sinuses | wrong outcome |
| Osaguona | 2017 | Bilaterally impacted permanent mandibular canines: A report of two cases. | wrong study design |
| Otsuka | 2001 | A review of clinical features in 13 cases of impacted primary teeth | wrong study design |
| Ozbey | 2024 | Awareness of patients with impacted teeth about impacted teeth in Turkey: A questionnaire study | wrong outcome |
| Paine | 2020 | Tooth developmental biology: disruptions to enamel-matrix assembly and its impact on biomineralization | wrong outcome |
| Pallikaraki | 2014 | Developmental dental anomalies assessed by panoramic radiographs in a Greek orthodontic population sample | wrong outcome |
| Papadopoulou | 2019 | Palatal canine impaction is not associated with third molar agenesis | wrong outcome |
| Papagiannis | 2017 | Orthodontic treatment of a patient with dentin dysplasia type I and bilateral maxillary canine impaction: Case presentation and a family-based genetic analysis | wrong study design |
| Parsons | 2021 | Improved taste and texture in novel popcorn varieties compared to conventional lines | wrong outcome |
| Patel | 2024 | Assessment of maxillary canine impaction and severity in young patients. | wrong outcome |
| Paul | 2024 | A rare case of compound odontome with impacted tooth: Understanding clinico-radio-histopathological aspects | wrong outcome |
| Peck | 1994 | The palatally displaced canine as a dental anomaly of genetic origin | wrong study design |
| Pedulla | 2015 | Endodontic surgery of a deviated premolar root in the surgical orthodontic management of an impacted maxillary canine. | wrong outcome |
| Pei | 2024 | The crucial role of centrioles in tooth growth and development | wrong population |
| Pell | 1942 | Report on a ten-year study of a tooth division technique for the removal of impacted teeth | wrong outcome |
| Pelsmaekers | 2016 | The genetic contribution to dental maturation | wrong outcome |
| Pemberton | 2007 | Microscale genetic differentiation in a sessile invertebrate with cloned larvae: investigating the role of polyembryony | outcome |
| Peng | 2022 | Optimization design for dynamic characteristics of face gear drive with surface-active modification | wrong outcome |
| Perera | 2024 | Genetic variation in apolipoprotein A-V in hypertriglyceridemia | wrong outcome |
| Perina | 2023 | Two new species of Atopobathynella (Parabathynellidae, Bathynellacea) from the Pilbara region, Australia | wrong population |
| Peterson | 1998 | Principles of management of impacted teeth | wrong outcome |
| Pillai | 2015 | Incidence of agenesis, impactions, angular positions and pathologies related to third molar teeth | wrong outcome |
| Pimper | 2010 | Mitochondrial DNA variation and population structure of Commerson's dolphins (Cephalorhynchus commersonii) in their southernmost distribution | wrong population |
| Pinho | 2017 | Developmental disturbances associated with agenesis of the permanent maxillary lateral incisor | wrong outcome |
| Pogrel | 2003 | Transplantation of impacted teeth | wrong outcome |
| Precious | 1998 | Presence of impacted teeth as a determining factor of unfavorable splits in 1256 sagittal-split osteotomies | wrong outcome |
| Primo | 2011 | Impacted teeth: new diagnostic perspectives | wrong outcome |
| Puthiyaveetil | 2020 | Epithelial - mesenchymal interactions in tooth development and the significant role of growth factors and genes with emphasis on mesenchyme - A review | wrong study design |
| Putri | 2024 | Characteristics of radiolucent lesions associated with impacted teeth at RSGM Unpad | wrong outcome |
| Quilodrán | 2018 | Effect of hybridization with genome exclusion on extinction risk | wrong outcome |
| Quirynen | 2000 | Periodontal health of orthodontically extruded impacted teeth. A split mouth, long term clinical evaluation | wrong outcome |
| Racek | 2009 | Heredity of canine teeth retention | wrong outcome |
| Racek | 2008 | Personal views on the heredity of the retention of canine teeth | wrong study design |
| Rahman | 2013 | Analysis of the immunoexpression of Ki-67 and Bcl-2 in the pericoronal tissues of impacted teeth, dentigerous cysts and gingiva using software image analysis | wrong outcome |
| Rai | 2009 | Accuracy of BR regression equation for impacted teeth in age estimation in Haryana population of India | wrong outcome |
| Rai | 2009 | Effect of nutrition on coronal displacement of cementum in impacted teeth | wrong outcome |
| Rai | 2020 | Non-syndromal multiple supernumerary and permanent impacted teeth in mother and her one child | wrong study design |
| Rasmussen | 2013 | Inherited primary failure of eruption in the primary dentition: report of five cases | wrong study design |
| Razpet | 2007 | Re-evaluation of Salmo data by Gridelli (1936) - Description of stocking, hybridization and repopulation in the River Soca basin | wrong outcome |
| Reed | 2024 | Ecological impact of american chestnut hybrid restoration on invertebrate communities above- and belowground | wrong outcome |
| Reis | 2018 | Impact of FokI (rs2228570) and BglI (rs739837) polymorphisms in VDR gene on permanent tooth eruption: A cross-sectional study | wrong outcome |
| Ren | 2021 | Genotype-phenotype pattern analysis of pathogenic PAX9 variants in Chinese Han families with non-syndromic oligodontia | wrong outcome |
| Retrouvey | 2012 | Dental development and maturation, from the dental crypt to the final occlusion | wrong outcome |
| Rhoads | 2019 | Establishing the diagnostic criteria for eruption disorders based on genetic and clinical data | wrong outcome |
| Riva-Rossi | 2020 | Revalidation of the Argentinian pouched lamprey Geotria macrostoma (Burmeister, 1868) with molecular and morphological evidence | wrong population |
| Rogerson | 1993 | Infections associated with impacted teeth | wrong outcome |
| Rohani | 2021 | Correlation between maxillary cuspid impaction with available space and anomalies of maxillary lateral incisors. | wrong outcome |
| Romoli | 1987 | Alopecia areata and homolateral headache due to an impacted superior wisdom tooth | wrong outcome |
| Rounds | 1933 | A consideration of anesthesia for impacted teeth | wrong outcome |
| Rowe | 2009 | Women who are well informed about prenatal genetic screening delay emotional attachment to their fetus | wrong outcome |
| Ruangchan | 2024 | Genetic variants in KCTD1 are associated with isolated dental anomalies | medical condition |
| Ruangchan | 2020 | Genetic variants in KCTD1 are associated with isolated dental anomalies | wrong outcome |
| Rudick | 2019 | Understanding the pathophysiology behind chairside diagnostics and genetic testing for IL-1 and IL-6 | wrong outcome |
| SaÄŸlam | 2003 | Clinical and radiologic investigation of the incidence, complications, and suitable removal times for fully impacted teeth in the Turkish population. | wrong outcome |
| Sadrhaghighi | 2022 | Evaluation of the association of alveolar bone dimensions in unilateral palatally impacted canine: A cone-beam computed tomography analysis. | wrong outcome |
| Sajnani | 2012 | Early prediction of maxillary canine impaction from panoramic radiographs. | wrong outcome |
| Sajnani | 2015 | Dental anomalies associated with buccally- and palatally-impacted maxillary canines | medical condition |
| Salman | 1958 | The problem of impacted teeth | wrong outcome |
| Salvolini | 1997 | Two and three-dimensional computed tomography in the diagnosis of impacted teeth | wrong outcome |
| Saunders | 2003 | Nasal aspergillosis associated with an impacted canine tooth in a Belgian Shepherd dog | wrong population |
| Sawamura | 2003 | Impacted teeth in the maxilla: usefulness of 3D Dental-CT for preoperative evaluation | wrong outcome |
| SchoenebeckB | 2019 | Molecular characterization of human impacted third molars: diversification of compartments | wrong outcome |
| Sekiya | 2015 | A case of impacted tooth in the maxillary sinus: CT findings | wrong outcome |
| Senkumar | 2023 | Hox genes and its application in dentistry | wrong outcome |
| Shapira | 2003 | Intrabony migration of impacted teeth | wrong outcome |
| Shapira | 2014 | Mandibular second molar impaction. Part I: Genetic traits and characteristics | wrong outcome |
| Sharma | 2015 | A Versatile Spring Design for Management of Impacted Teeth in Anterior Arch | wrong outcome |
| Sharma | 2010 | Coronal displacement of cementum in impacted teeth and its correlation to age: A preliminary study | wrong outcome |
| Sharma | 2016 | Failure of eruption of permanent molars: a diagnostic dilemma. | wrong outcome |
| Shastri | 2014 | A newer simultaneous space creation, eruption, and adjacent root control spring for the management of impacted tooth | wrong outcome |
| Shpack | 2022 | The presence of impacted teeth (except for wisdom teeth) in orthodontic patients in israel | wrong outcome |
| Šidlauskienė | 2016 | Genetic and environmental impact on variation in the palatal dimensions in permanent dentition: A twin study | wrong study design |
| Sim | 2004 | The eruption guidance of impacted maxillary anterior teeth | wrong outcome |
| Singh | 2011 | Inverted and impacted teeth: Report of four rare cases | wrong study design |
| Singh | 1996 | Management of asymptomatic impacted wisdom teeth: A multicentre comparison | wrong outcome |
| Skurikhina | 2018 | Phylogeography and demographic history of the Pacific smelt Osmerus dentex inferred from mitochondrial DNA variation | wrong outcome |
| Smardz | 2024 | The importance of genetic background and neurotransmission in the pathogenesis of the co-occurrence of sleep bruxism and sleep-disordered breathing-review of a new perspective | wrong study design |
| Smith | 2008 | An evaluation of the association between three -dimensional cranial morphology and molecular distances in humans | wrong outcome |
| Sofer | 2019 | A fully adjusted two-stage procedure for rank-normalization in genetic association studies | wrong outcome |
| Solinski | 2019 | Synthetic simplification of carolacton enables chemical genetic studies in streptococcus mutans | wrong outcome |
| Song | 2023 | Study of the transmission characteristics of the cycloid gear based on a multi-objective optimization modification | wrong outcome |
| Spern | 2024 | Retention of neutral genetic diversity and connectivity within the metapopulation of Florida Scrub-Jay at Kennedy Space Center | wrong outcome |
| Square | 1982 | Blues reinstate impacted teeth benefit in 1983 FEHBP | wrong outcome |
| Stanaitytė | 2014 | Tooth size discrepancies and dental arch width in patients with palatally and labially impacted maxillary canines | wrong outcome |
| Stoltz | 2023 | High genetic diversity in American chestnut (Castanea dentata) despite a century of decline | wrong population |
| Sun | 2023 | Knowledge, attitudes, and practice of non-medical students regarding impacted teeth: A cross-sectional study | wrong outcome |
| Sun K | 2019 | Functional study of novel PAX9 variants: The paired domain and non-syndromic oligodontia | wrong outcome |
| Švalkauskienė | 2016 | Heritability estimates of dental arch parameters in Lithuanian twins | wrong outcome |
| Tanaka | 2008 | An adolescent patient with multiple impacted teeth | wrong outcome |
| Tatli | 2009 | Autotransplantation of impacted teeth: A report of 3 cases and review of the literature. | wrong study design |
| Taylor | 2022 | Blue crab Callinectes sapidus dietary habits and predation on juvenile winter flounder Pseudopleuronectes americanus in southern New England tidal rivers | wrong population |
| Thonusin | 2020 | The impact of genetic polymorphisms on weight regain after successful weight loss | wrong outcome |
| Thotakura | 2024 | A review of contributing factors to heart failure: genetic, lifestyle, and environmental influences | wrong study design |
| Tian | 2014 | The relationship between maxillary anterior impacted teeth and sagittal facial type | wrong outcome |
| Tomaszewska | 2015 | Is the area of the orbital opening in humans related to climate? | wrong outcome |
| Townsend | 2016 | Genetic aspects of dental disorders | wrong outcome |
| Tsuji | 2015 | A case of ameloblastic fibro-odontoma accompanied with a number of impacted teeth | wrong outcome |
| Turska | 2021 | Knowledge and attitude of polish dental healthcare professionals during the COVID-19 pandemic | wrong outcome |
| Twyana | 2021 | Knowledge of impacted teeth among the undergraduate dental students of a medical college: a descriptive cross-sectional study | wrong outcome |
| Tymofiyeva | 2010 | Three-dimensional localization of impacted teeth using magnetic resonance imaging | wrong outcome |
| UGA | 1971 | Two cases of the multiple impacted teeth on maxilla and mandible | wrong study design |
| Ullah | 2022 | Analysis of a discrete stator hybrid excited flux switching linear machine | wrong outcome |
| Valle | 2013 | Diversidade genetica de populares naturais de pariparoba [Pothomorphe umbellata (L. ) Miq.] por RAPD | wrong outcome |
| Vatani | 2022 | Study of a modular toothed linear hybrid reluctance motor with permanent magnets in translator slots | wrong outcome |
| Vermette | 1995 | Uncovering labially impacted teeth: apically positioned flap and closed-eruption techniques | wrong outcome |
| Vinjolli | 2017 | Dental anomalies in an Albanian orthodontic sample: a retrospective study | wrong outcome |
| Walker | 2016 | Genetic deletion of the clathrin adaptor gga3 reduces anxiety and alters GABAergic Transmission | wrong outcome |
| Wan | 2022 | Investigation on the dent rebound mechanism and the structural multi-objective optimization of sandwich pipes | wrong outcome |
| Wang | 2005 | Comparison of dentomaxillary pantomography and periapical radiographs with horizontal tube shift in localizing the impacted teeth | wrong outcome |
| Wang | 2017 | ClC-7 deficiency impairs tooth development and eruption | wrong population |
| Wang | 2016 | Detection and functional analysis of BMP2 gene mutation in patients with tooth agenesis | medical condition |
| Wes | 2011 | Impacted maxillary cuspid. I. Etiology and diagnosis | wrong study design |
| Westerlund | 2019 | Epigenetic markers of tooth eruption - DNA methylation and histone acetylation | wrong outcome |
| Wolujewicz | 1980 | Fractures of the mandible involving the impacted third molar tooth: an analysis of 47 cases | wrong study design |
| Wong | 1985 | Maintaining an ideal tooth-gingiva relationship when exposing and aligning an impacted tooth | wrong outcome |
| Xu | 2021 | Evolutionary trends in human mandibles and dentition from Neolithic to current Chinese | wrong outcome |
| Yamamoto | 2003 | Diagnostic value of tuned-aperture computed tomography versus conventional dentoalveolar imaging in assessment of impacted teeth | wrong outcome |
| Yamamoto | 2002 | Tuned-Aperture Computed Tomography (TACTÂ®) to assess impacted teeth using 2D slices and 3D pseudo-holograms | wrong outcome |
| Yamaoka | 1996 | Completely impacted teeth in dentate and edentulous jaws | wrong outcome |
| Yamaoka | 1995 | Influence of adjacent teeth on impacted third molars in the upper and lower jaws | wrong outcome |
| Yan | 2013 | Etiologic factors for buccal and palatal maxillary canine impaction: A perspective based on cone-beam computed tomography analyses. | wrong outcome |
| Yang | 2023 | Adaptive grinding method and experimental verification of worm gear tooth surface knife marks | wrong outcome |
| Yesiltepe | 2022 | Evaluation the relationship between the position and impaction level of the impacted maxillary third molar teeth and marginal bone loss, caries and resorption findings of the second molar teeth with CBCT scans | wrong outcome |
| Yildirim | 2004 | Multiple impacted permanent and deciduous teeth | wrong outcome |
| Yu | 2011 | Goals and guidelines for residency training in cytopathology. | wrong outcome |
| Yudistiro | 2023 | Optimization and innovative design of dental implants under dynamic finite element analysis | wrong outcome |
| Zam | 2024 | Management of submandibula abscess dextra et causa impaction teeth at Sultan Fatah Hospital Demak | wrong outcome |
| Zanatta | 2013 | Phylogeography and genetic variability of the freshwater mussels (Bivalvia: Unionidae) Ellipse, Venustaconcha ellipsiformis (Conrad 1836), and Bleeding Tooth, V. Pleasii (Marsh 1891) | wrong population |
| Zanelli M | 2024 | Co-occurrence of JAK2-V617 F mutation and BCR:ABL1 translocation in chronic myeloproliferative neoplasms: a potentially confounding genetic combination | wrong outcome |
| Zeitler | 1993 | Management of impacted teeth other than third molars | wrong outcome |
| Zhang | 2021 | Comparison of the gingival thickness, keratinized and attached gingival widths of unilateral labially and palatally impacted maxillary canines erupted by closed eruption technique | wrong outcome |
| Zhang | 2024 | Contact stress reliability analysis model for cylindrical gear with circular arc tooth trace based on an improved metamodel | wrong outcome |
| Zhang | 2021 | Optimization of modular SPM machines considering stator modularity | wrong outcome |
| Zhen | 2019 | Uprighting deeply impacted mandibular second molars with miniscrew anchorage | wrong outcome |
| Zhou | 2021 | Transcriptome sequencing of gingival tissues from impacted third molars patients reveals the alterations of gene expression | wrong outcome |
| Zhu | 2013 | Longitudinal genetic effects on mandibular position of female twins from six to twelve years old | wrong study design |
| Ziegler | 1980 | Impacted tooth ligation chain | wrong outcome |
| Zuccati | 2014 | Multiple bilateral impactions in an adolescent girl | wrong study design |
| Zulian | 2017 | Correlation dental classification of mandibular impacted tooth with a duration of action odontectomy in rumkital Dr. Ramelan Surabaya | wrong outcome |
